# Reactivity and selectivity modulation within a molecular assembly: recent examples from photochemistry

**DOI:** 10.1007/s43630-021-00146-3

**Published:** 2021-12-16

**Authors:** Yeshua Sempere, Martin Morgenstern, Thorsten Bach, Manuel Plaza

**Affiliations:** Department Chemie and Catalysis Research Center (CRC), Technische Universität München, 85747 Garching, Germany

**Keywords:** photochemistry, supramolecular, molecular assembly, organic synthesis

## Abstract

In recent years, photochemical reactions have emerged as powerful transformations which significantly expand the repertoire of organic synthesis. However, a certain lack of selectivity can hamper their application and limit their scope. In this context, a major research effort continues to focus on an improved control over stereo- and chemoselectivity that can be achieved in molecular assemblies between photosubstrates and an appropriate host molecule. In this tutorial review, some recent, representative examples of photochemical reactions have been collected whose unique outcome is dictated by the formation of a molecular assembly driven by non-covalent weak interactions.

**Graphical Abstract:**

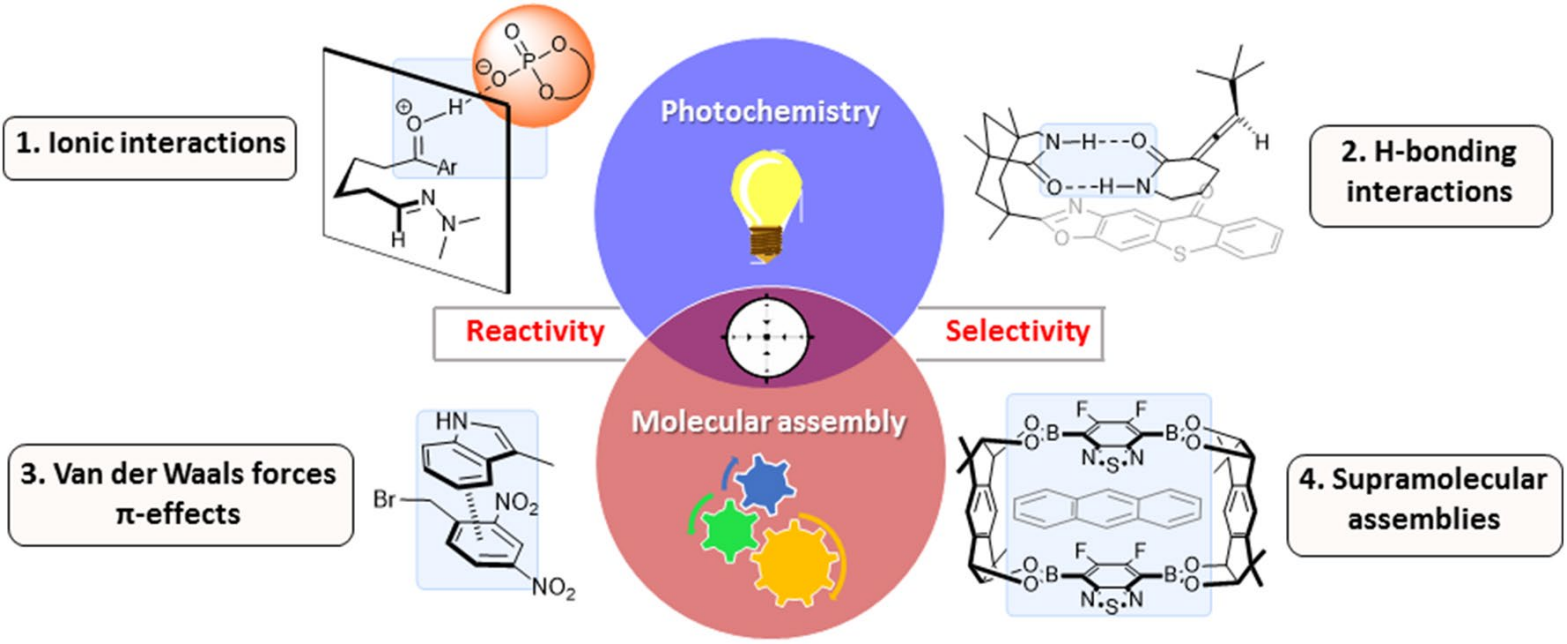

## General Introduction

Sunlight is the most abundant and safest source of energy that is provided to us. Nature has employed it as a source of energy for the development and creation of the world as we know it. As we move away from fossil fuels towards a greener and more environmentally friendly society, light will continually grow in importance as an unlimited supply of energy. In the field of organic synthesis, the first attempts to use light as an energy source date back to the nineteenth century [1]. The way organic chemists use light has experienced tremendous development since then [2]. Nowadays, hot topics in photochemistry involve, for instance, the synthesis of molecules with high optical purity, the exploration of new reactivity pathways, the revalorization of feedstock materials, etc. For these purposes, not only substrates and light are required, but in most cases also extra elements which provide the necessary spatial arrangement or help in the energetic transfer for the desired transformation to occur [3]. In this Tutorial Review, we aim to highlight different kinds of non-covalent weak interactions that promote the formation of molecular assemblies of the substrates with these extra elements, which in turn dictate the overall outcome of a given photochemical transformation [4, 5]. In the present document, we have selected some of the latest examples on how different assemblies of components in a reaction medium can modulate the reactivity or the selectivity of the system [6]. The concept of an assembly is understood herein as a specific and reversibly formed molecular construction that is held together by intermolecular non-covalent interactions or weak covalent bonds (metal coordination). These assemblies present photochemical properties, e.g. excited state lifetimes that differ from those present in the substrate [7, 8]. Therefore, the formation of the assembly significantly affects and modulates the course and selectivity of a given photoreaction [9]. The nature of the assembly is determined by several factors, including structure, charge, or electronic state of the interacting species as well as the solvent, temperature, additives, and other parameters. However, the discussion of assemblies in which the reaction medium plays a significant role in a supramolecular construction are not covered here. Furthermore, the formation of organometallic complexes is also understood as an assembly process of organic substrates to a metal centre in a reversible manner. The review has been organized according to the individual interactions responsible for assembly formation, namely the following: ionic interactions, hydrogen bond interactions, van der Waals forces and π-effects, and multiple interactions in supramolecular assemblies. The main purpose of this review was to illustrate the most recent and profound developments in the field in a non-comprehensive manner.

## Ionic interactions

Different types of assemblies can be constructed by means of strong and weak ionic interactions. In this section, we illustrate with different examples the most general types of ionic interactions. This section has been divided into three subsections depending on the strength and type of bond generated between substrate and catalyst. The first subdivision includes metal-substrate coordination, in which the organic substrate and the metal centre are physically linked by a coordination bond. The next subsection contains the general ionic interaction between a cation and an anion, both organic and inorganic. The final subsection covers acid/base interactions, which includes both Brønsted and Lewis acids and their interactions with organic photosubstrates.

### Metal coordination

In this first subsection, we have included recent examples of assemblies between metal complexes and substrates, which normally bind through a heteroatom. The photochemical properties of these assemblies differ from their individual components, the metal and the substrate. Thus, the assembly can be irradiated selectively, which allows for chemo- or stereoselective reactions.

**Scheme 1:**
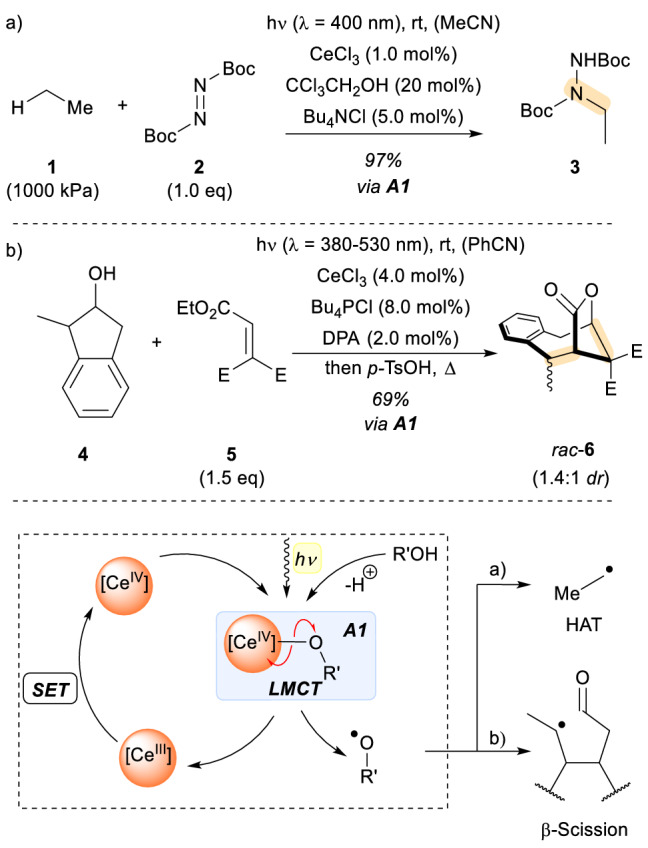
**a** Cerium catalysed functionalization of inert alkanes by a photoredox process. **b** Photochemical oxidation of alkoxides, β scission, and alkene coupling. *E* electron withdrawing group, *DPA* 9,10-diphenylanthracene, *SET* single electron transfer, *HAT* hydrogen atom transfer

Cerium is an example of a metal that has recently found increased applications in the field of organic photocatalysis. The use of this relatively abundant metal for the alkylation, arylation or C − H amination of alkanes, including methane and ethane, was reported by the group of Zuo [10]. The strategy developed by the authors involved the assembly of a cerium(IV) salt with an alcohol, e.g. trichloroethanol, generating a cerium-alkoxy metal complex (Scheme 1). The irradiation of this assembly triggers the ligand-to-metal charge transfer (LMCT), leading to the homolytic cleavage of the oxygen-metal bond and generating an electrophilic alkoxy radical and a reduced Ce(III) salt. The alkoxy radical is responsible for the abstraction of a hydrogen atom from the substrate of the reaction, in this case ethane (**1**). While the alcohol is regenerated, the alkyl radical reacts with the provided coupling partner, in the case of C − H amination DBAD (di-*tert*-butyl azodicarboxylate, **2**). Addition of the alkyl radical to aromatic rings or Michael acceptors is also possible [11]. After the coupling, the formed *N*-centered radical is reduced by the Ce(III) salts regenerating the required Ce(IV) and delivering the reaction product. Most impressively, the alkoxy radical selectively reacts with the highly stable C − H bond of ethane (bond dissociation energy BDE 439 kJ/mol) in the presence of the more reactive acetonitrile C − H (BDE 389 kJ/mol). The authors explain this phenomenon by polarity matching effects, meaning that the electrophilic alkoxy radical preferentially reacts with a more electron-rich C − H bond of a compound like eethane than an electron poor one of a similar bond-strenght. The reaction with acetonitrile would instead generate another electrophilic radical and is hence disfavoured [12]. After disclosure of this method for simple alkane functionalization, the same approach was recently applied to more complex organic molecules. As shown in Scheme 1b), this strategy can be extended to cycloalkanols such as **4** which, after assembly with the Ce(IV) salt and upon irradiation with visible light, form a highly reactive alkoxy radical via LMCT. The radical undergoes β-bond scission generating an alkyl radical, which can add to Michael acceptors such as **5**. The resulting acceptor-substituted radical undergoes single electron reduction by 9,10-diphenylanthracene (DPA) to create a nucleophilic enolate that completes the formal cycloaddition reaction by an intramolecular aldol reaction. In the example shown in Scheme 1, the bridged lactone *rac*-**6** was obtained in 69% yield. While the generation of alkoxy radicals from alcohols is photochemically difficult since the redox potential of alkoxides E_1/2_(R’O^•^/R’O^−^) is highly positive, the formation of the assembly with cerium enables this process and the subsequent chemistry.

**Scheme 2:**
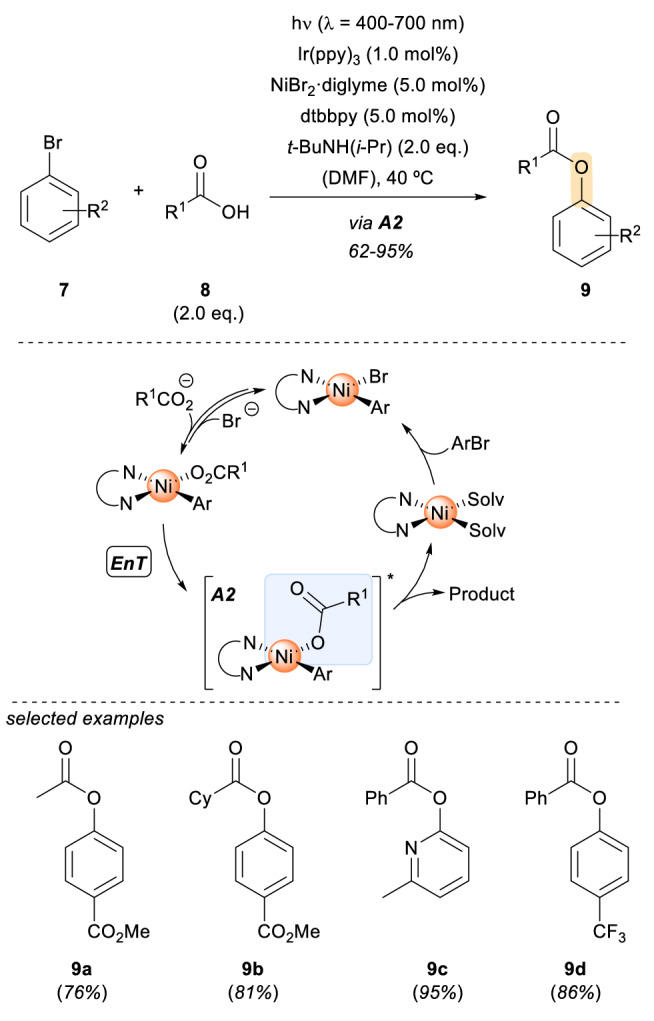
Nickel-catalysed photoenabled cross-coupling of aryl halides with carboxylic acids. *Ppy* 2-phenylpyridine, *dtbbpy* 4,4’-di-*tert*-butyl-2,2’-bipyridine, *diglyme* bis(2-methoxyethyl)ether

Another common metal in traditional thermal catalysis that has found its way into photoredox catalysis is nickel [13]. Recently, the group of MacMillan has shown the viability of excited-state organometallic catalysis via direct photoexcitation using a Ni catalyst [14]. With the development of this method, the authors provide a tool for the coupling of carboxylic acids **8** with aryl halides **7** (Scheme 2). A wide array of *O*-arylated esters were obtained in good to excellent yields (**9a**-**9d**). The scope included primary, secondary, and tertiary alkyl carboxylic acids as well as benzoic acid derivatives with different electron densities of the aromatic ring. A large selection of differently substituted aryl bromides was also successfully implemented. Mechanistically, the catalytic cycle was suggested to start with an oxidative addition of the aryl bromide to the Ni(0) catalyst, in this case dtbbpy·Ni(0). Subsequently, the Ni(II) complex performs a ligand exchange from the bromide to the carboxylate substrate, pre-organizing all the necessary partners for the cross-coupling reaction. Once the assembly **A2** is formed, energy transfer (EnT) from the triplet state of Ir(ppy)_3_, the photosensitizer, to the nickel complex occurs. The electronically excited Ni(II) complex undergoes reductive elimination to generate the corresponding *O*-arylated esters. The formation of **A2** proved to be crucial for bringing together the coupling partners as well as for enabling the corresponding cross-coupling reaction. The authors’ data support an initial Dexter energy transfer to the Nickel complex rather than a photoredox pathway via single electron transfer (SET). Photosensitization mechanisms which give access to excited-state organometallic catalysts have not been deeply explored yet. As this mechanism is not strongly established, alternative mechanisms involving two-photon cascades and a Ni(III) species are postulated [15, 16].

**Scheme 3:**
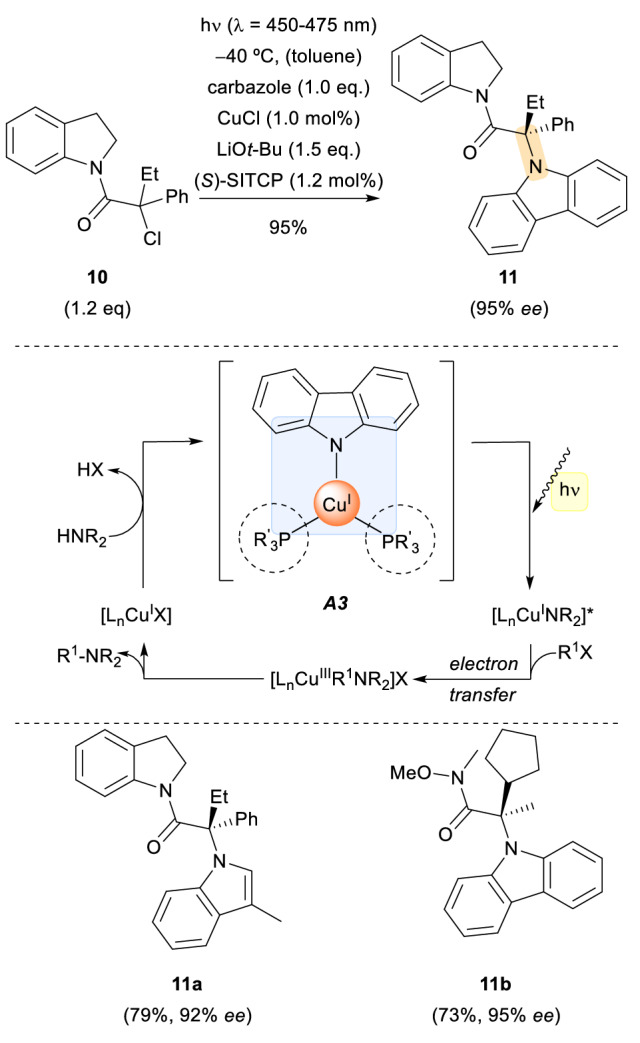
Copper-catalysed enantioselective C − N cross-coupling reaction under visible light irradiation. *(S)-SITCP* (11*aS*)-( +)-5,6,10,11,12,13-hexahydro-5-phenyl-4H-diindeno[7,1-cd:1,7-ef]phosphocin

The last highlighted example in the metal-coordination subsection concerns the highly abundant and environmentally benign element copper, which has an ever-growing impact in visible-light photoredox catalysis, especially in stereoselective metallaphotoredox transformations [17]. In a report from 2016, the Fu group described a photoinduced Cu-catalysed enantioconvergent coupling of racemic tertiary alkyl chlorides **10** with amines such as carbazole (Scheme 3) [18]. They employed Cu(I) salts in combination with a chiral phosphine ligand (R’_3_P: (*S*)-SITCP) and nucleophilic amines, assembling a photochemically active metal complex. The corresponding products are amines, mostly *N*-substituted carbazoles and indoles, with a newly formed stereocentre in the α-position to the carbonyl group (**11a-11b**). The products were obtained in high enantioselectivity, despite the fact that the phosphine ligand was used in relatively low quantities compared to the abundant and competitive ligand, the amine nucleophile (see Scheme 3). Mechanistically, the reaction between the amine and the alkyl halide was suggested to occur via an inner sphere pathway. To support their proposal, the authors synthesized the complex **A3** separately and employed it in place of CuCl and R’_3_P under the same reaction conditions. The product was obtained in comparable yields and optical purity, with no coupling occurring in the absence of light. Therefore, these experiments supported the notion that the intermediate metal complex is catalytically active. It is noteworthy that the complex **A3** itself is photochemically active, meaning that no external photoredox complex is necessary, and a racemic background reaction is prevented.

### Cation-Anion interactions

When considering assemblies formed by classical ionic interactions, the most obvious interaction is that of a negatively charged substrate with a positively charged molecular entity to form a ground state complex (or vice versa). Although literature precedence for the use of these types of assemblies in supramolecular photochemistry is rather scarce, there are still a few examples which nicely prove the usefulness and efficacy of these reactions.

**Scheme 4:**
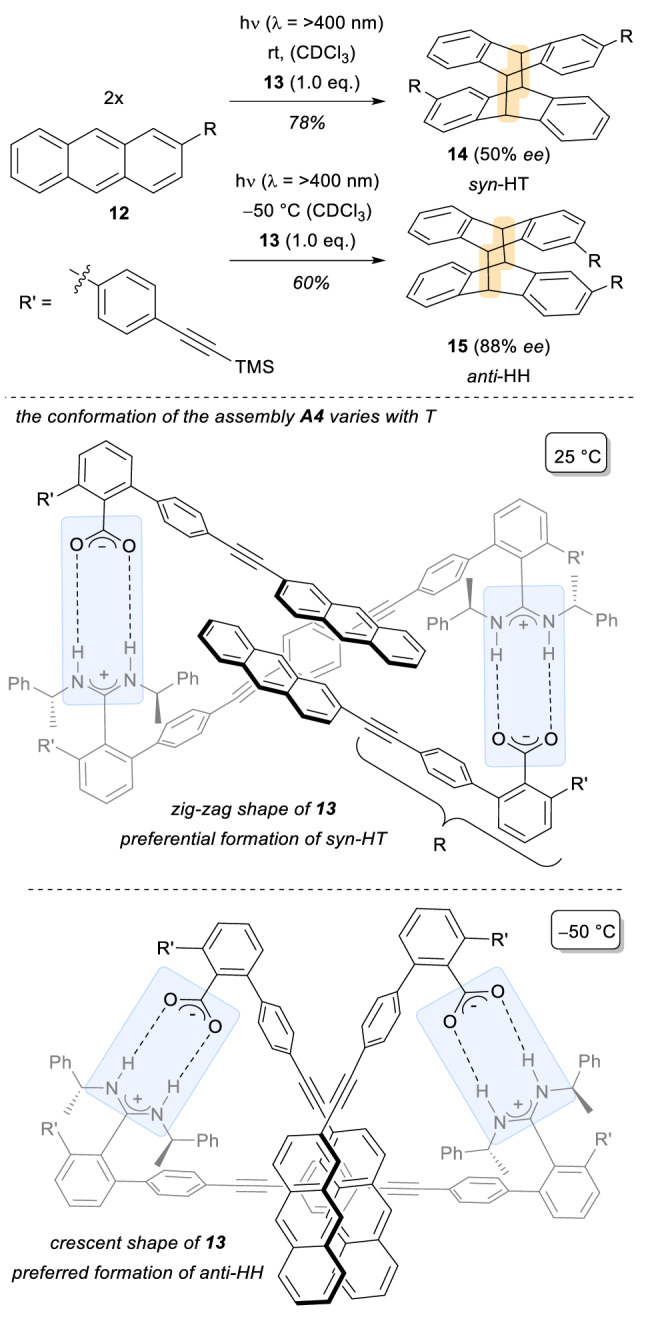
Carboxylate/amidinium salt ionic interactions in the stereoselective photodimerization of anthracenic acids **12**

In this context, the work reported by Yashima and co-workers in 2017 is particularly interesting [19]. Therein, amidinium-carboxylate salt bridges enabled an example for successful template-directed [4 + 4] photocycloadditions of prochiral anthracene derivatives **12**. The regioselectivity of the transformation was dictated by conformational changes of the chiral template **13** at different temperatures, which either features a zig-zag shape for the assembly (25 °C) or a crescent shape (− 50 °C), as depicted in Scheme 4.

As illustrated in Scheme 4, due to the ionic interactions between the template and anthracenes **12**, the conformation of the overall assembly **A4** granted the exclusive formation of either the *syn*-HT (head-to-tail) isomer **14** or the *anti*-HH (head-to-head) isomer **15**, respectively. Remarkably, as a consequence of the assembly formation, this reaction stands as the first example described in the literature of a template-controlled photodimerization of anthracenes driven by cation–anion interactions, which features high levels of regio-, diastereo- and enantioselectivities. Moreover, the value of 88% *ee* obtained in the formation of the *anti*-HH isomer **15** is the highest reported so far for hydrogen-bond/ionic template-assisted photodimerizations.

**Scheme 5:**
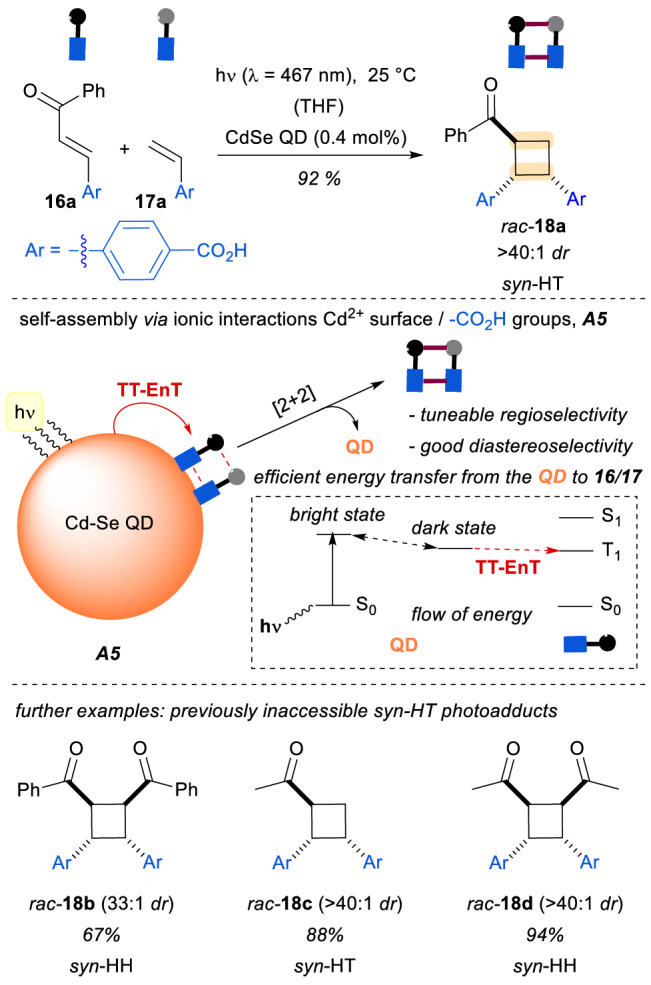
Ionic interactions between quantum dots and cinnamates **16** and **17** enabled an unprecedented formation of *syn*-HT isomers *rac*-**18**

Quantum photoinitiators have found recent applications in the field of supramolecular photochemistry [20]. The group of Weiss reported fundamental work on the use of quantum dots for an intermolecular stereoselective [2 + 2] photocycloaddition reaction of cinnamates **16** and styrene derivatives **17** [21]. The molecular assembly **A5** features a very efficient triplet-to-triplet energy transfer (TT-EnT) from the excited dark state of the quantum dot (Scheme 5), due to reversible ionic interactions between the cationic surface of the CdSe quantum dot and the carboxylic groups of the photosubstrates. The assembly allows for an exquisite control of the regio- and diastereoselectivity of the reaction, giving rise to the previously inaccessible *syn*-cyclobutane products *rac*-**18**. Overall, this approach overcomes the classical lack of control in the relative configuration of the cyclobutane products, where the formation of the *syn* isomer was highly disfavoured due to the stereoelectronic properties of the photosubstrates, and the corresponding *anti*-isomers predominated.

### Acid-Base interactions

**Scheme 6:**
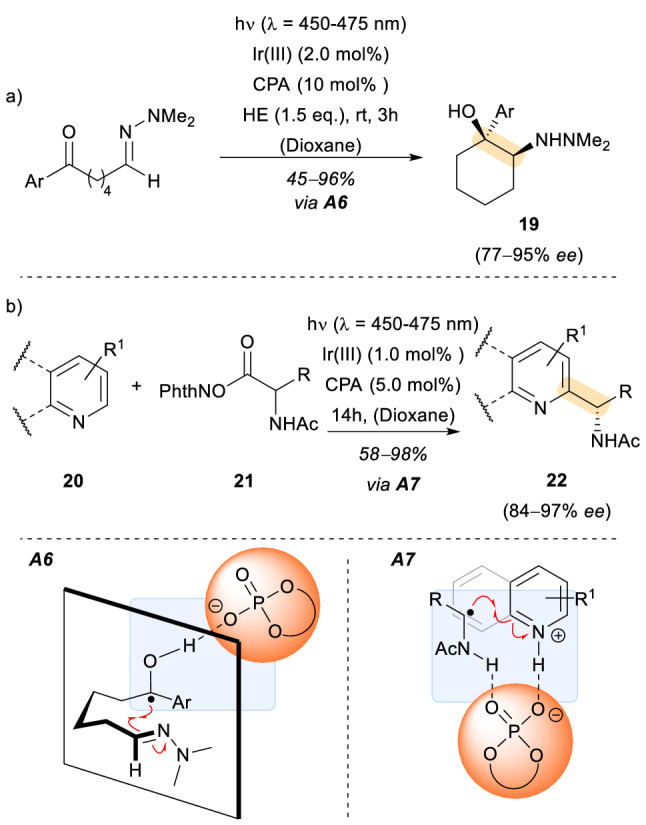
Chiral phosphoric acid catalysed radical photo C–C bond formation in cyclization reactions and Minisci-type reactions. HE: Hantzsch Ester; [Ir(III)]: **a** [Ir(ppy)_2_(dtbpy)]PF_6_, **b** [Ir(dF(CF_3_)ppy)_2_(dtbpy)]PF_6_; *CPA* chiral phosphoric acid, **a** *MacMillan TiPSY catalyst* (*R*)-3,3′-Bis(triphenylsilyl)-1,1′-binaphthyl-2,2′-diyl hydrogenphosphate, **b** *(R)-TRIP* (R)-3,3′-Bis(2,4,6-triisopropylphenyl)-1,1′-binaphthyl-2,2′-diyl hydrogenphosphate or *(R)-TCYP* (11bR)-4-Hydroxy-2,6-bis(2,4,6-tricyclohexylphenyl)-4-oxide-dinaphtho[2,1-d:1,2-f][1,3,2]dioxaphosphepin

Acid and base interactions have been employed in photochemical reactions, as well as in other fields of organic chemistry, to effectively control the enantioselectivity of a given transformation. In 2013, the group of Knowles reported an illustrative example of how a chiral Brønsted acid could be employed to control the spatial orientation of the different functional groups involved in an intramolecular photoreaction [22]. In this particular case, and based on previous experience of the research group, a ketyl radical was formed upon electron transfer from an iridium photoredox catalyst. In principle, a proton transfer from the corresponding chiral Brønsted acid (Scheme 6, A6) enabled the formation of the ketyl radical. The assembly created by a non-covalent association between the newly formed ketyl radical and the chiral phosphoric acid (CPA) provided the basis for the subsequent enantioselective, intramolecular aza-pinacol coupling. Moreover, it facilitated the access to protonated ketyl radicals at potentials (≈ 1 V) less reducing than the reduction potential of the parent ketone. Under optimized reaction conditions, several substituted and heterocyclic aromatic groups and different chain lengths were tolerated, providing the corresponding amino alcohols **19** in good yields and enantioselectivities.

More recently, the Phipps research group implemented a related strategy to achieve an enantioselective addition of α-amino alkyl radicals to heterocycles in a Minisci-type reaction [23]. In a separate photoredox step, one electron transfer from an in situ generated Ir(II) species forms the nucleophilic radical species. This Ir(II) complex is the resulting product of the prior single electron oxidation/rearomatization of the heterocycle, after the reduction by the excited Ir(III) catalyst. Eventually, the initial prochiral radical species which also bears a nitrogen atom forms the supramolecular assembly in association with the CPA and the heterocyclic substrate (Scheme 6, A7). This chiral supramolecular complex dictates the stereochemical outcome of the process. The reaction showed a broad scope for the redox active ester **21** as well as for the corresponding pyridines or quinolones **20** employed as substrates. In general, the Minisci-type products **22** were obtained in good yields and high enantioselectivities. Several observations made by the authors shed some light on the mechanistic pathway. For example, a binding point for the generation of a hydrogen bond between the redox active ester and the phosphoric acid was proven to be required. In the absence of such functional groups the reaction proceeded with negligible stereocontrol. Additionally, the observation of a non-linear relationship between the heterocyclic product and the optical purity of the catalyst suggested that a second molecule of chiral phosphoric acid catalyst might assist as a phosphate base.

**Scheme 7:**
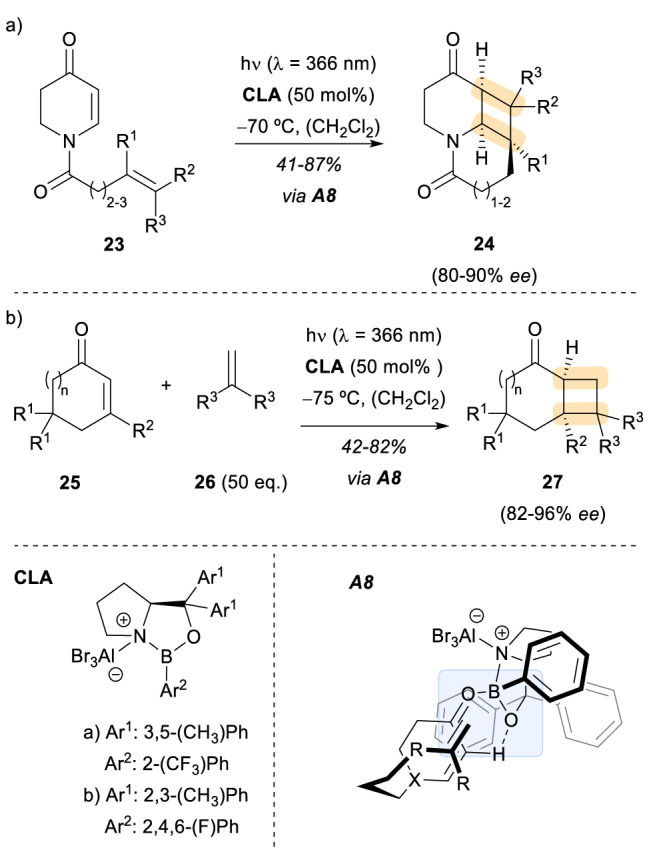
Intra- and Intermolecular enantioselective [2 + 2] photocycloaddition catalysed by an oxazaborolidine-based chiral Lewis acid: (*CLA*)

The use of chiral acids as catalysts for enantioselective photochemical reactions is not limited to Brønsted acids. In fact, the use of chiral Lewis acids (CLA) in combination with different photosubstrates, frequently α,β-unsaturated carbonyl compounds such as **23**, has been one of the most prolific fields of photocatalysis in the past years (Scheme 7) [24]. A remarkable example for the use of a CLA in an enantioselective photocycloaddition reaction was reported by the group of Bach in 2013 [25]. In this particular case, the formation of a Lewis acid-enone substrate complex **A8** allowed for selective irradiation with a distinct light source that excites preferably the catalyst-substrate assembly (higher absorption coefficient) in the presence of non-complexed substrate. With this strategy, the influence of the background reaction was lowered; nevertheless, its impact remained noticeable as seen from the high catalyst loading (50 mol%). This phenomenon could be explained by looking at the intersystem crossing (ISC) rates for the enone substrates. The required ISC from S_1_ ππ* to T_1_ ππ* for the catalysed reaction is rather slow, making the background reaction that proceeds via ISC from the S_1_ nπ* state competitive. Therefore, to guarantee products with high optical purity high Lewis acid loadings were needed. Indeed, with 50 mol% Lewis acid loading, in this case an oxazaborolidine-AlBr_3_ complex (Scheme 7, CLA-a), the photocycloaddition products **24** were obtained in moderate to good yields and high enantioselectivity, showing their utility as key intermediates in the synthesis of lupin alkaloids. Some years later, the same strategy was applied to the intermolecular and enantioselective [2 + 2] photocycloaddition reaction of typical cyclic α,β-unsaturated carbonyl compounds such as 2-cyclohexenone with olefins **26** (Scheme 7) [26]. This method delivered the cycloaddition products **27** in a similar yield and optical purity range as in the previous example. The chiral Lewis acid was, as in the earlier case, an oxazaborolidine-AlBr_3_ complex but with a different aromatic substitution pattern (Scheme 7, CLA-b). The ease in modifying the substituents of this Lewis acid complex has popularized its use as a catalyst for stereoselective photoreactions. The observed stereochemical outcome of these two photocycloaddition reactions is in agreement with the postulated assembly **A8** shown in Scheme 7. As depicted, regardless of the [2 + 2] photocycloaddition variant, the approach of the olefin to the alkene of the carbonyl compound occurs on the *Si*-face of the enone ring.

**Scheme 8:**
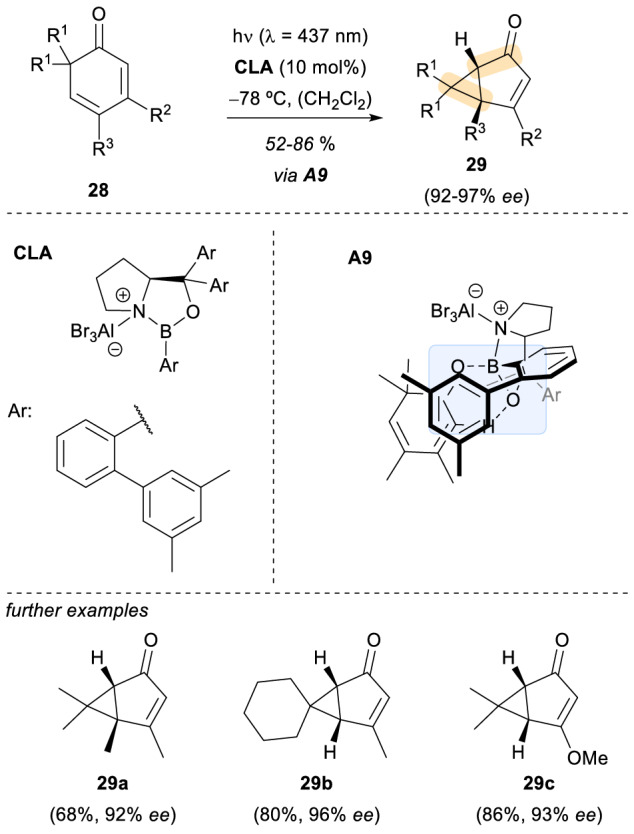
Enantioselective [2 + 2] photocycloaddition on the S_1_ state catalysed by an oxazaborolidine-based chiral Lewis acid complex

Additionally to T_1_ state reactions, the oxazaborolidine-AlBr_3_ complex has been employed as a chiral Lewis acid catalyst in reactions that occur from the S_1_ state. An illustrating example was reported in 2019 by the Bach group employing substituted 2,4-cyclohexadienones as substrates (Scheme 8, 28) [27]. As in previous uses of this catalyst, the irradiation of the complexed substrates **A9** can be carried out selectively. This selective irradiation prompted an asymmetric oxa-di-π-methane rearrangement to occur. The absorption profile of the complexed substrate showed a larger bathochromic shift than that of previous enone substrates. This behaviour suppressed any potential uncatalysed background reactions. Furthermore, the absence of competing reactions allowed a lower Lewis acid loading in comparison with previous reports. The substrate scope of this transformation included different functional groups for the substituents R^1^, R^2^ and R^3^ such as aliphatic chains bearing olefins, ethers, aromatic groups or halogen atoms (Scheme 8, 29a-29c). An enantioselective synthesis of *trans-*chrysanthemic acid was also carried out using the aforementioned strategy. In addition, quantum chemical calculations employing density functional theory (DFT) supported the hypothesis that the reaction takes place on the singlet potential energy surface. The computational tools revealed that a model substrate-Lewis acid complex (with BF_3_ as the Lewis acid) was able to reach the S_1_ state as a result of an allowed ππ* transition.

**Scheme 9:**
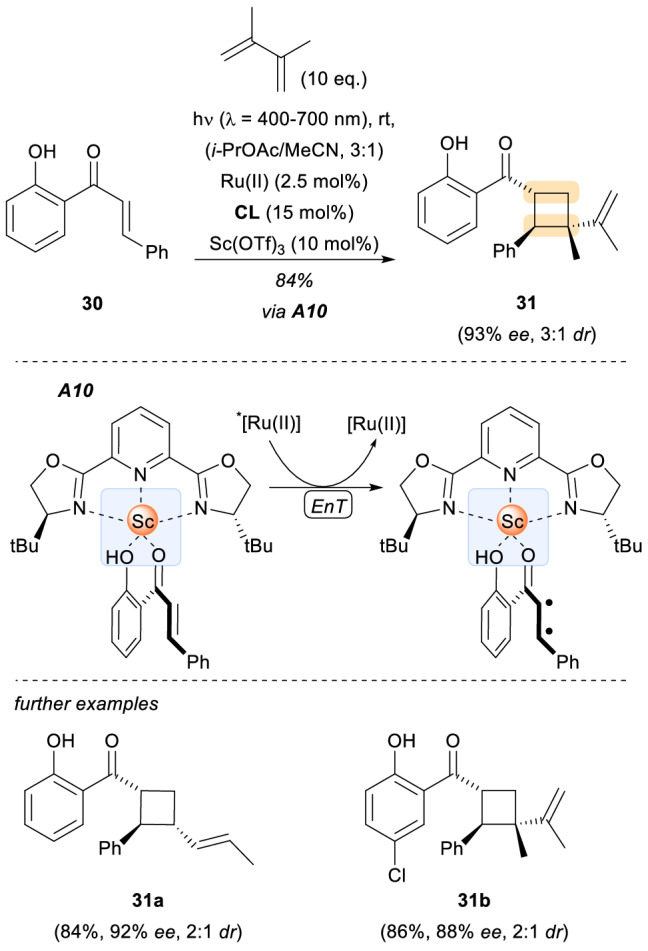
Enantioselective [2 + 2] photocycloaddition catalysed by a scandium-based CLA complex. *CL* chiral ligand/*t*-BuPyBox, *Ru(II)* [Ru(bpy)_3_](PF_6_)_2_

Chiral Lewis acids have not been employed with direct irradiation exclusively but also in combination with a photocatalyst. In 2014, the research group of Yoon reported a dual catalysis strategy consisting of a ruthenium-based photosensitizer and a chiral europium complex as the cocatalyst [28]. With this work, the authors showed that a Lewis acid could control the stereochemical outcome of a reaction initiated by photoinduced electron transfer from an electronically excited photocatalyst. Based on this premise, an asymmetric [2 + 2] photocycloaddition of α,β-unsaturated ketones was developed. A few years later, the same group described the discovery of a scandium-based chiral Lewis acid complex that catalysed triplet energy transfer from an electronically excited photosensitizer [29]. They applied this to an asymmetric [2 + 2] photocycloaddition of 2′-hydroxychalcones **30** with different dienes (Scheme 9, 31a-31b). Computational calculations of the S_0_-T_1_ difference supported the hypothesis that catalyst-substrate assembly **A10** had an energetically lower T_1_ state than the free chalcone substrate **30**. These computational predictions were corroborated by measurements of the emissive properties of the substrate **30** at near-infrared wavelengths in the presence and the absence of Sc^3+^. In addition, the emission band measured in the presence of Sc^3+^ was partially quenched when oxygen was present, consistent with a T_1_ emission.

**Scheme 10:**
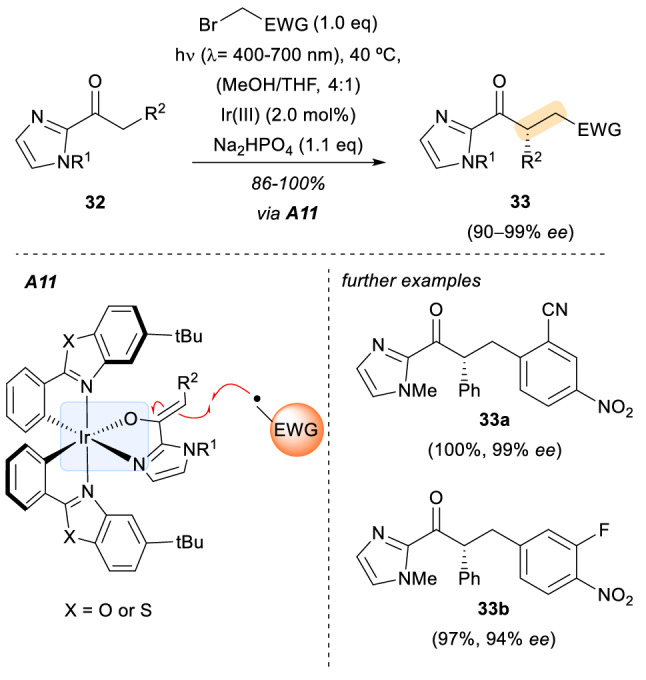
Enantioselective α-alkylation of 2-acyl imidazole substrates by electron-poor benzyl bromides under visible light irradiation. *EWG* electron withdrawing group, *Ir(III)* structure shown in **A11** but with two molecules of MeCN in place of the substrate

In parallel, the group of Meggers established that the previous dual catalysis strategy could be condensed to the use of only one chiral metal complex [30]. Essentially, a chiral transition metal complex that served as a photoredox catalyst and a source of stereochemical information during the reaction was found. Two octahedral chiral-at-metal Ir(III) complexes, previously synthetized by the Meggers group for the activation of α,β-unsaturated 2-acyl imidazoles **32** as electrophiles, served the purpose. In the presence of these metal catalysts, the α-alkylation of 2-acyl imidazole substrates by electron-poor benzyl bromides occurred in high yields and optical purity (Scheme 10, 33a-33b). It should be noted that here the Ir(III) complex does not affect the triplet energy of the substrate, instead it acts as a typical photoredox catalyst that generates the electrophilic radical species by SET. The authors emphasize the importance of the assembly **A11** which controls the stereochemical outcome of the electrophilic radical addition to the electron rich metal-coordinated enolate, and at the same time functions as the in situ generated chiral photosensitizer. The substrate–catalyst assembly **A11** is formed by the displacement of the labile acetonitrile ligands from the original Ir(III) complexes.

## Hydrogen bond interactions

Hydrogen-bonding templates have perhaps stood out in the last decade as the most widely reported catalysts for light-driven reactions enabled by weak interactions [31]. In this case, normally two well-defined, directional hydrogen bonds between the template and a photosubstrate create a molecular assembly that allows for original transformations to occur. In this context, a H-bond driven formation of both exciplexes and ground-state complexes are covered in this section.

Over the past decade, several research groups have employed chiral H-bonding templates to achieve enantioselective photochemical transformations. In this regard, the formation of a catalyst–-substrate assembly would enable enantioselective transformations to take place within the chiral environment of the photocatalyst, which normally involves a triplet energy transfer from the catalyst to the photosubstrate.

This strategy has been applied successfully to achieve a wide variety of enantioselective [2 + 2] photocycloadditions [32, 33]. More recently, several photochemical deracemization protocols have emerged as powerful and novel methodologies to create chirality in an unprecedented manner [34]. For this reason, we will focus in this section on the most recent and significant examples of this kind of transformation.

**Scheme 11:**
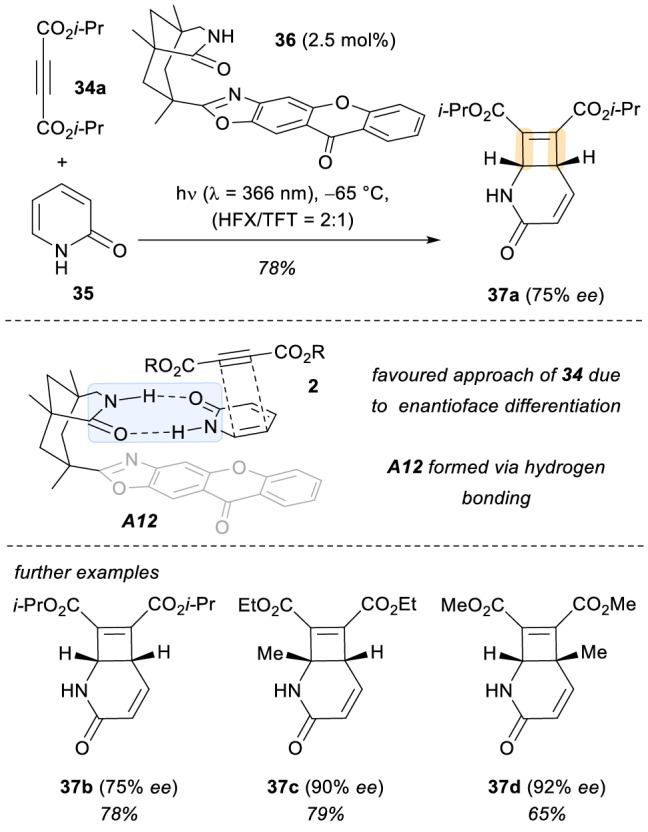
Template control enantioselective [2 + 2] photocycloadditions using a chiral xanthone **3** as sensitizer. *HFX* hexafluoroxylene, *TFT* α,α,α-trifluorotoluene

In 2014, the group of Bach reported the first example of a catalytic template-controlled intermolecular [2 + 2] photocycloaddition (Scheme 11) [35]. Herein, a chiral xanthone catalyst **36** was employed [36]. This molecule presents two different moieties: a lactam motif that allows for a two-point H-bond coordination of the photosubstrate, and a xanthone core which is responsible for the triplet energy transfer to **35** within the chiral environment of the photocatalyst. In this context, once the complex **A12** is formed, an enantioface differentiation enables a selective addition of the alkynes **34**, and the corresponding final products **37** were obtained with very good enantioselectivities (up to 92% *ee*).

**Scheme 12:**
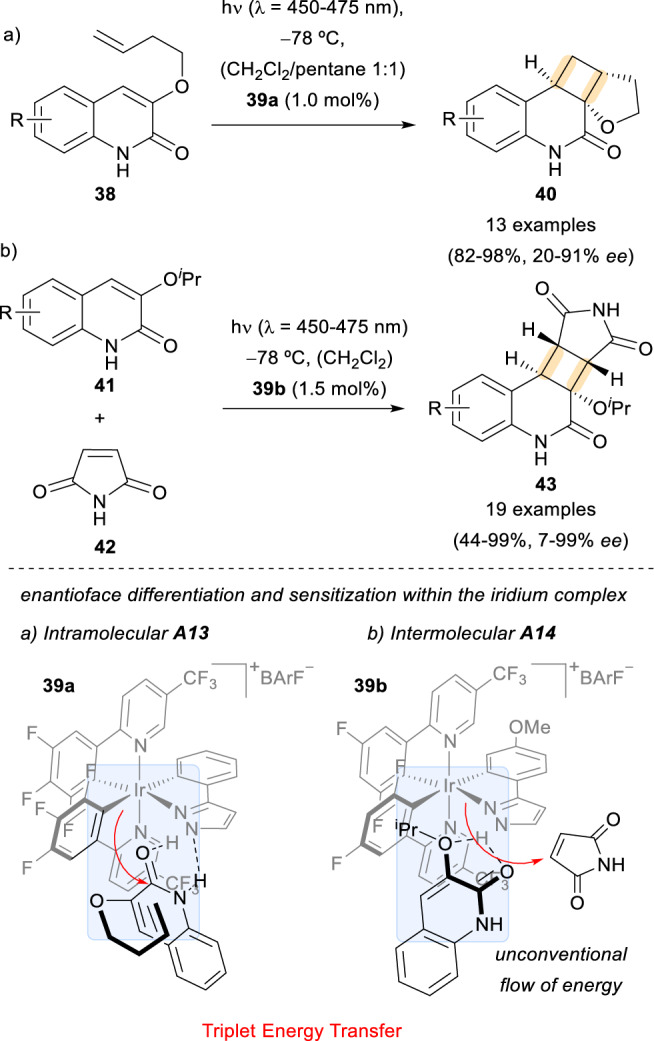
Intra- and intermolecular photocycloadditions employing iridium complexes with an H-bonding site as sensitizers

In general, enantioselective photochemical reactions mediated by triplet energy transfer from a sensitizer have experienced an outstanding growth in the past years. After the previously mentioned study, other template-controlled catalytic enantioselective [2 + 2] photocycloadditions were developed by other groups. Of particular interest is the work of Yoon and co-workers on iridium-based templates containing an H-bonding motif. Their first contribution featured the intramolecular version of this kind of transformation (Scheme 12, a) [37]. There, the iridium metal complex **39a** acted as a triplet sensitizer and formed an assembly **A13** due to H-bonding interactions between its pyrazolyl residue and the quinolones **38**, creating a chiral environment for a subsequent cycloaddition to take place with very good levels of enantioselectivity to form the final products **40**.

Much more challenging was the intermolecular approach of these reactions (Scheme 12, b) [38]. Remarkably, upon photoexcitation of the assembly **A14** (formed in a similar fashion to the previous example **A13**), there is an unconventional energy flow from sensitizer **39b** to the electron-poor alkene **42** instead of the bound substrate **41**. Once again, the reaction showed high levels of efficiency both in terms of yields and enantiomeric excesses in the formation of the photoproducts **43** (up to 99% *ee*).

**Scheme 13:**
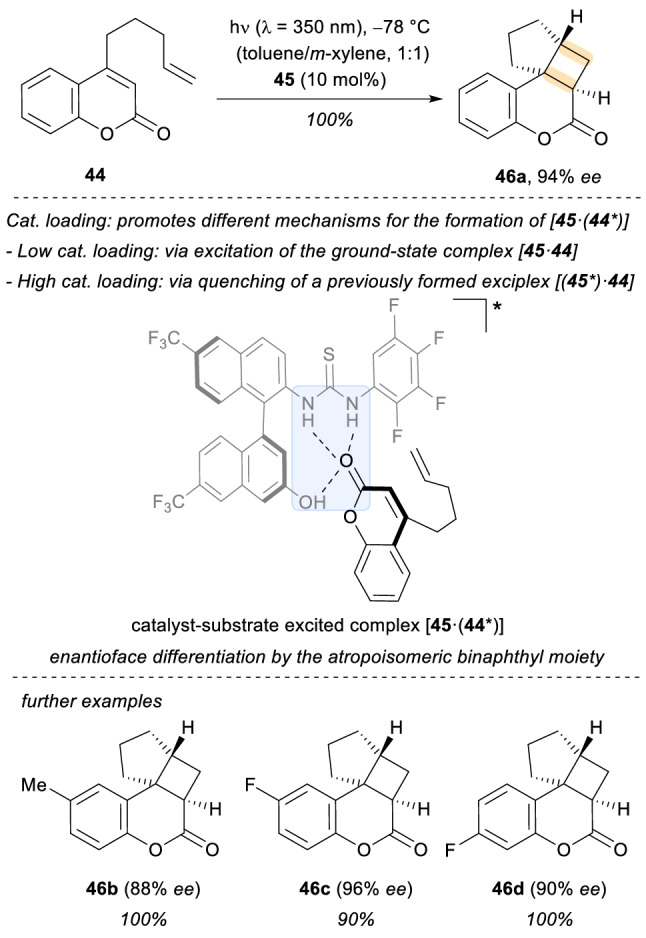
Enantioselective intramolecular [2 + 2] photocycloadditions enabled by chiral thiourea templates

An alternative and original strategy for enantioselective intramolecular [2 + 2] photocycloadditions was reported by the group of Sivaguru in 2016 based on the employment of chiral thiourea templates (Scheme 13) [39, 40]. In contrast to previous examples, this approach does not rely on the classical energy or electron transfer from a catalyst to a photosubstrate once the assembly is formed. Specifically, atropoisomeric binaphthyl-based chiral thioureas **45** formed here an assembly via H-bonding to the quinolone substrates **44**. Mechanistically, the nature of the light-absorbing species depends on the amount of catalyst. If a low catalyst loading is present, the transformation presumably takes place though direct excitation of the ground-state complex **44·45**, thanks to a bathochromic shift in the absorption that enables its selective excitation at λ = 350 nm, to end up forming 45·(44*). On the other hand, at high loading of catalyst, the higher optical density of **45** promotes its selective excitation to end up being quenched by **44** to access again the complex 45·(44*), but this time, via the formation of a (45*)·44 exciplex.

In both scenarios, the final formation of the excited complex 45·(44*) is responsible for the delivery of the final products **46** with very good enantioselectivity (up to 96% *ee*).

**Scheme 14:**
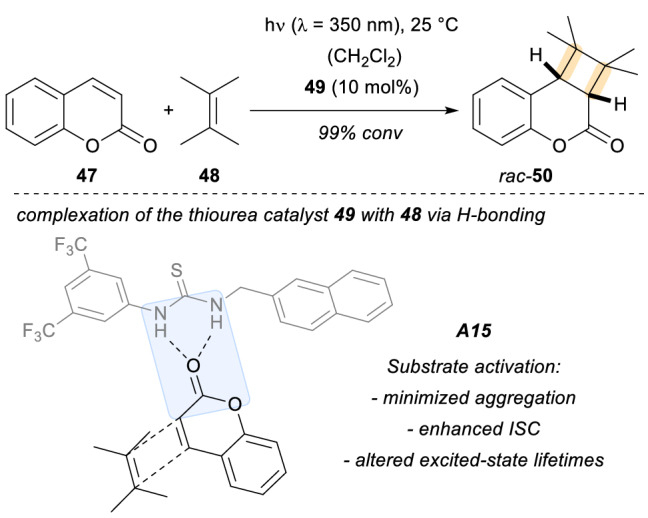
Intermolecular [2 + 2] photocyclizations of coumarins and alkenes employing achiral thiourea templates

Two years later, the same group extended the scope of their work with thiourea templates to an example of achiral intermolecular [2 + 2] photocycloadditions employing quinolones **47** with olefins such as **48** (Scheme 14) [41]. The reaction proceeded with quantitative yield to form the final photoproduct *rac*-**50**. From a mechanistic point of view, the implementation of thiourea template **49** to form the assembly **A15** allowed for minimized aggregation (preventing the formation of dimers of the photosubstrate), enhanced the inter-system crossing rates, and altered the excited-state lifetimes of **47**.

As stated in the introduction of this section, light-driven deracemization methods have been very recently reported. In a deracemization reaction, one of the two enantiomers of a starting racemic mixture is selectively converted into the other one. Given that such processes are thermodynamically disfavoured (∆S < 0), the success of these reactions on a thermal manifold appears not possible. However, photochemistry offers a solution to circumvent this problem, given that the loss in entropy can be compensated by light, which is the driving force of these transformations.

**Scheme 15:**
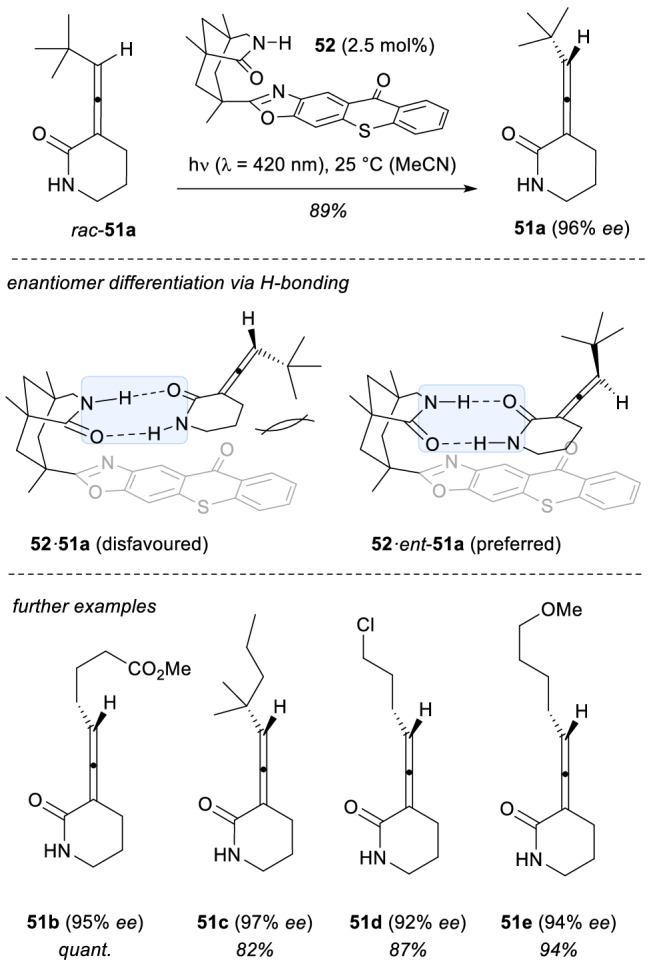
Photochemical deracemization of allenes *rac-51a* using a chiral thioxanthone template **52** as the sensitizer

In this context, in the end of 2018 the group of Bach reported the first example of a photochemical deracemization reaction (Scheme 15) [42, 43]. This strategy was proven to be very efficient for an enantioselective access to allenes **51** (89–97% *ee*). Mechanistically, a chiral photosensitizer **52** provides an enantiodifferentiation in the H-bonding of the allene *ent*-**51** (favoured) vs. **51** (disfavoured) due to steric repulsions of the residue at the terminal carbon of the allene, which can be pointing down or up. In this way, the favoured assembly **52·ent**-**51** featured a more efficient triplet energy transfer from the thioxanthone core of the photocatalyst than the analogous complex **52·51** upon irradiation at λ = 420 nm. The selective excitation of *ent*-**51** racemizes this allene forming statistically **51** and *ent*-**51** in equal amounts. Eventually, as a consequence of the iterative selective racemization of *ent*-**51** by the photocatalyst, the starting racemic allenes are converted into the enantiomers **51** with very high levels of efficiency. Remarkably, the same photochemical deracemization strategy could be successfully extended to other photosubstrates, such as chiral cyclopropanes [44]. and sulfoxides [45].

**Scheme 16:**
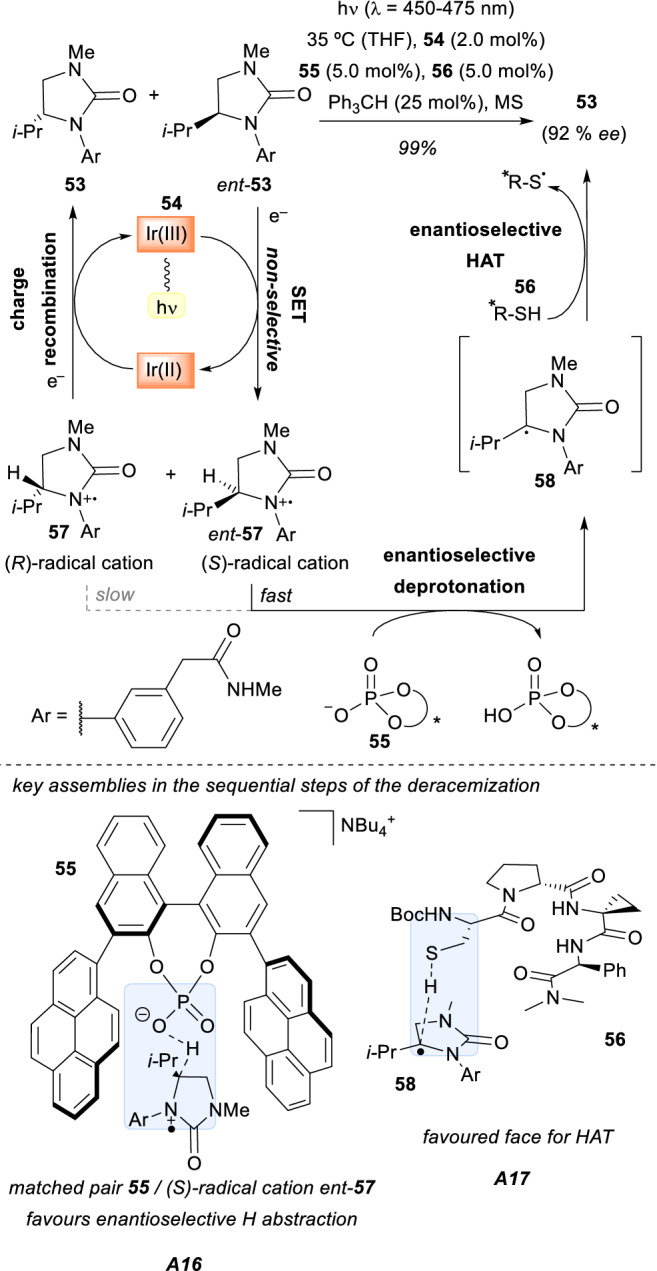
Light-driven photoredox deracemization of cyclic urea substrates. *Ir(III)* [Ir(dF(CF_3_)ppy_2_)(bpy)]PF_6_

Another important example of a light-driven deracemization was reported in 2019 by Miller, Knowles, and co-workers [46]. Their work was based on the deracemization of cyclic ureas **53** under visible light conditions (Scheme 16). The mechanism involved a complex sequence of steps that is depicted in Scheme 16. Starting with an excited-state Ir(III) chromophore **54**, a non-selective single electron transfer generates the racemic radical cation species **57** and *ent*-**57**. Subsequently, a chiral phosphate base **55** enantioselectively deprotonates the (*S*)-radical cation *ent*-**57** through the formation of the **A16** assembly to afford a planar intermediate **58**.

At this point, the (*R*)-radical cation **57** evolves through charge recombination to regenerate compound **53**. Eventually, an enantioselective HAT from a chiral peptide **56** to intermediate **58** within the assembly **A17** reforms compound **53** with high optical purity. In this way, two synergistic chiral catalysts promote two different catalytic cycles with different key assemblies, but it is their particular combination that dictates the overall success in the high levels of enantioselectivities achieved in the deracemization of the starting racemic cyclic ureas **53** (up to 92% *ee*).

We would like to conclude this chapter by mentioning halogen bonds, which are another interesting variant for intermolecular interactions. Although they have been used in the field of supramolecular chemistry, to the best of our knowledge, they have not yet found their way into supramolecular photochemistry [47].

## Van der Waals forces and π-effects

Distance-dependent interactions between atoms or molecules such as van der Waals forces or π-effects also play an important role in the formation of supramolecular assemblies [48]. These molecular interactions are typically weaker, leaving their complexes more susceptible to changes in the media than the previously mentioned H-bonding or ionic interactions. One of the most relevant examples in photochemistry of an assembly between two molecules bound by π-effects are Electron Donor–Acceptor (EDA) complexes [49]. An electron-rich molecule and an electron-accepting molecule constitute these assemblies. Recently, the use of EDA complexes in photochemical transformations has been extensively reviewed by the group of Melchiorre [50]. Consequently, this Tutorial Review will cover a few illustrative examples of EDA complexes where π-effects are present in the formation of those complexes.

**Scheme 17:**
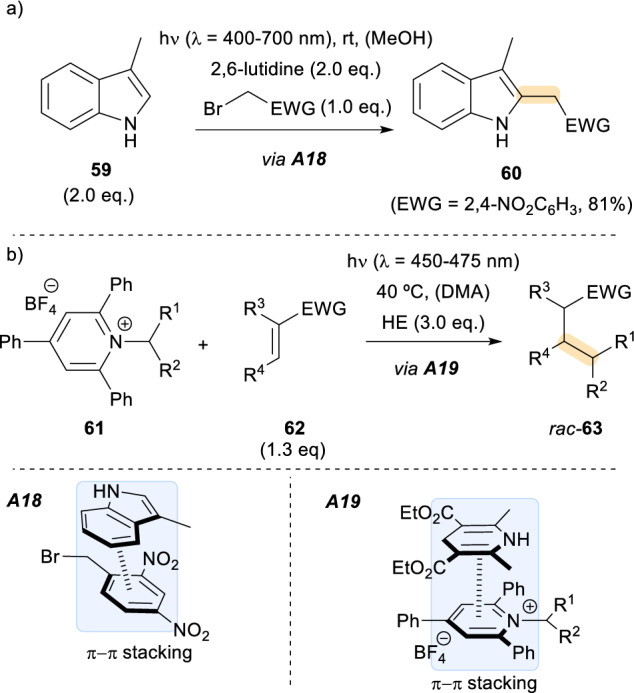
EDA complexes by π–π stacking. **a** Coupling of indoles with aryl bromides. **b** Radicals formed from Katritzky pyridinium salts coupled to alkenes. *EWG*: Electron Withdrawing Group; *DMA*: Dimethyl Acetamide; *HE*: Hantzsch Ester

In 2015, the group of Melchiorre reported a photochemical alkylation of 3-substituted indoles **59** (donors) with electron-poor benzyl and phenacyl bromides (acceptors) (Scheme 17, a) to generate the substituted indoles **60** in good yields. Interestingly, the authors observed that after mixing the solutions of the donor and the acceptor in methanol, the solution turned red. The colouring of the solution indicated that an assembly between the substrates was formed and showed a new absorption band in the visible light region. From a mechanistic point of view, the excitation of an EDA complex by light is followed by a single electron transfer from the donor to the acceptor. A radical ion pair is formed followed by the fragmentation of the C − Br bond in the acceptor. Then, the recombination of radicals occurs rapidly within the solvent cage, generating the desired alkylated products. However, what made this report exceptional is the fact that the authors were able to isolate and characterize the EDA complex **A18** by X-ray crystallography. The measurement of the interplanar distance between the 3-substituted indole and the electron-poor benzyl bromide in the crystalline framework turned out to be lower than the expected value for typical van der Waals interactions between aromatic molecules. The authors suggested that this is in accordance with intermolecular binding forces present in the solid state of this complex. Another example of π-effects and their application for EDA complexes is the deaminative functionalization described by the group of Aggarwal [51]. Katritzky pyridinium salts **61** and Hantzsch ester form, presumably due to π–π interactions, an EDA complex shown in Scheme 17 as **A19**. The irradiation of this assembly led to electron transfer, deamination and subsequently generation of non-stabilized alkyl radicals, which then underwent a plethora of transformations such as allylation, alkynylation or thioetherification reactions. It is worth mentioning that in this protocol the Hantzsch ester does take part in forming the EDA complex, but it is not the coupling partner, and instead acts as a sacrificial hydride donor and is stoichiometrically consumed without ending up in the final product.

**Scheme 18:**
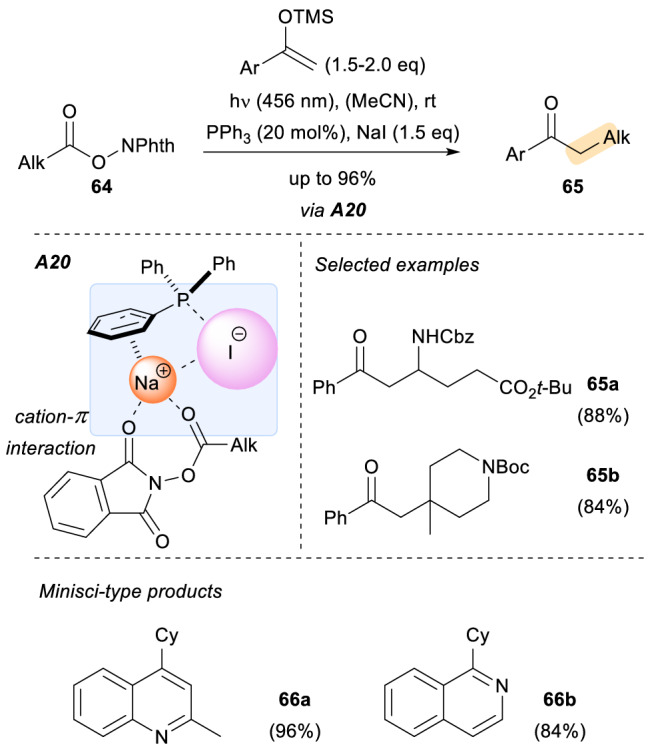
Generation of a photoactive three-component charge transfer complex for the generation of alkyl radicals

Shang and Fu recently reported the assembly of triphenylphosphine and sodium iodide together with redox active esters **64** to generate a photoactive three-component charge transfer complex (CTC, **A20**) [52]. The authors, supported by various computational calculations, postulated that the generation of a Ph_3_P-I• species after irradiation of the mixture was crucial for this process. As indicated by DFT calculations, not only was the iodide anion required in the formation of the CTC, but also the sodium cation, which plays an important role stabilizing the Ph_3_P-I• radical by cation-π interactions with the aromatic moiety of the phosphine [53]. Other alkali halides proved to be inferior for this purpose. The choice of triphenylphosphine for stabilizing the iodine radical is not trivial either, and as in the case of NaI, other phosphines led to less successful results. Under visible light irradiation of this three-component assembly, alkyl radicals are generated via decarboxylation of the parent activated esters. These alkyl radicals can then be trapped by silyl enol ethers (Scheme 18, 65a–65b) or acid activated pyridines and quinolones in a Minisci fashion (Scheme 18, 66a–66b). Moreover, the Minisci-type alkylations could be carried out in tandem with chiral phosphoric acids to provide the corresponding products in high enantioselectivity. These results are equivalent to the work by the Phipps group, previously mentioned in Sect. 2.3. In addition, the use of trisubstituted phosphines in combination with NaI can be employed for single electron transfer reductions in deaminative alkylation reactions using Katritzky *N*-alkylpyridinium salts and trifluoromethylation reactions using Togni’s hypervalent iodine reagent.

## Multiple interactions in supramolecular assemblies

The modulation of reactivity and selectivity of photoreactions by supramolecular assemblies can also involve a higher number of interactions between the substrate and the other partners/host of the assembly than in the previously described cases. Normally, these supramolecular assemblies require several host–guest weak interactions which assist in the pre-aggregation and spatial orientation of the photosubstrates. Several of the previously mentioned non-covalent interactions (H-bonding, van der Waals forces, acid/base, cation/anion interactions, π-effects) can work together to aid in modulating the reactivity of the system and/or its stereochemical outcome. The following cases will highlight some of the most representative and up-to-date examples of complex supramolecular assemblies employed in photocatalysis.

**Scheme 19:**
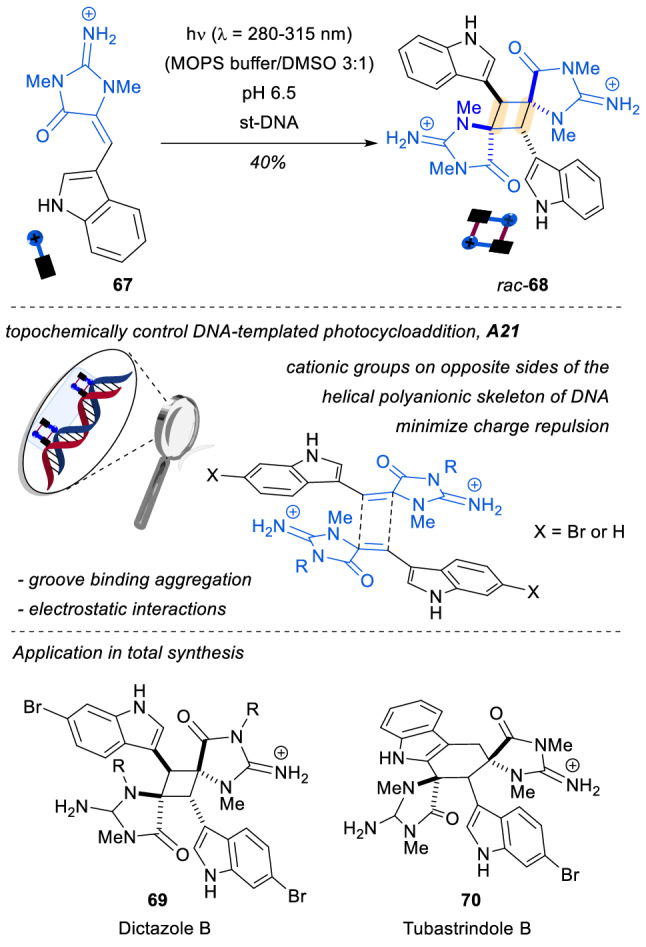
DNA-templated [2 + 2] photocycloadditions: first application in natural product synthesis: *st*-DNA: salmon testes DNA; *MOPS*: 3-(*N*-morpholino)propanesulfonic acid

Our first selected example of supramolecular assembly employed DNA as template and has been developed recently for the synthesis of dictazole-type cyclobutanes which can be used as key intermediates in the synthesis of natural products. It is remarkable that until 2018, all methods described in the literature for the synthesis of these compounds were restricted to solid-state synthesis. In that year, the group of Arseniyadis reported the first synthesis of dictazole-type compounds in solution thanks to a DNA-templated [2 + 2] photocycloaddition reaction [54]. The key assembly within the minor groove of the *st*-DNA of the two monomers of **67** is believed to result from the electrostatic interactions between the polyanionic skeleton of the DNA and the cationic groups of the individual monomers, which are oriented head-to-tail to minimize charge repulsion (Scheme 19). The formation of the assembly **A21** provides the reaction with good levels of regioselectivity in the formation of the spiro-fused cyclobutanes *rac*-**68**, due to an optimal orientation of the photosubstrates in the space within the DNA groove. Moreover, the molecules Dictazole B and Tubastrindole B (**69** and **70**, respectively) could be accessed thanks to this method, standing as the first example of a DNA-templated photoreaction that found application in the synthesis of natural products.

**Scheme 20:**
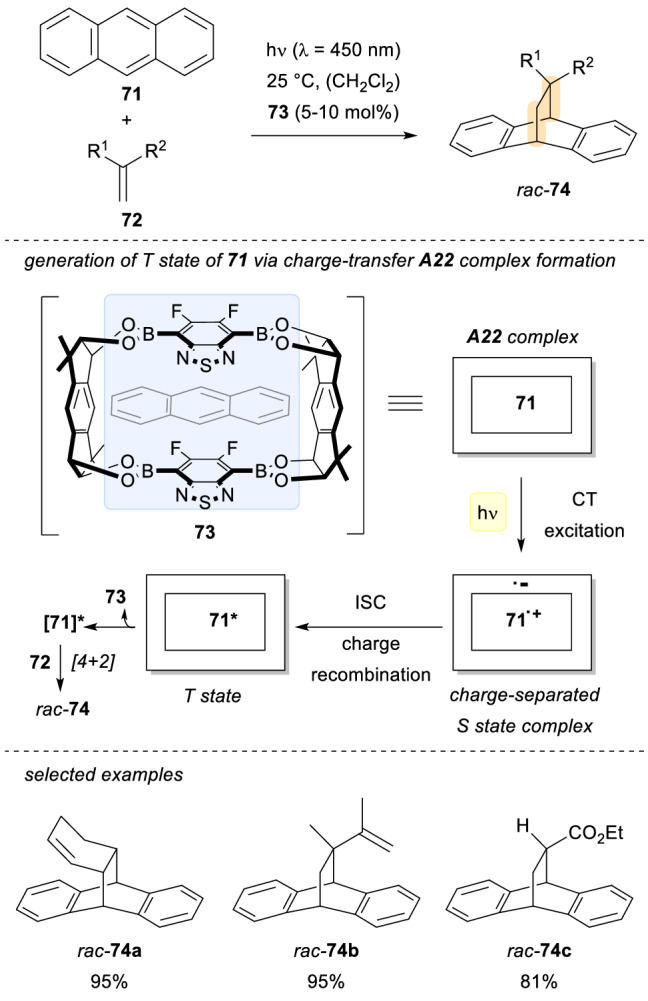
Charge-transfer complex formation and subsequent [4 + 2] photocycloaddition reactions

Another interesting type of supramolecular assemblies are those involving cages as host molecules [55]. Recently, the group of Iwasawa reported the use of a macrocyclic electron-deficient boronic ester for the in situ generation of the triplet state of anthracene via charge-transfer excitation in solution (Scheme 20) [56]. A supramolecular guest–host assembly of the anthracene within the boronic cage **A22** enabled the formation of the excited anthracene 71*. This excited substrate underwent subsequent [4 + 2] photocyclization reactions with different alkenes **72** to afford the final products **74** in very good yields. A donor–acceptor interaction between **71** and **73**, followed by radical ion pair formation and subsequent charge recombination are responsible for the generation of the excited anthracene. Remarkably, this transformation represents the first example described in the literature of a charge-transfer excitation due to an assembly in guest–host complexes that has an application in a catalytic C − C bond forming reaction.

**Scheme 21:**
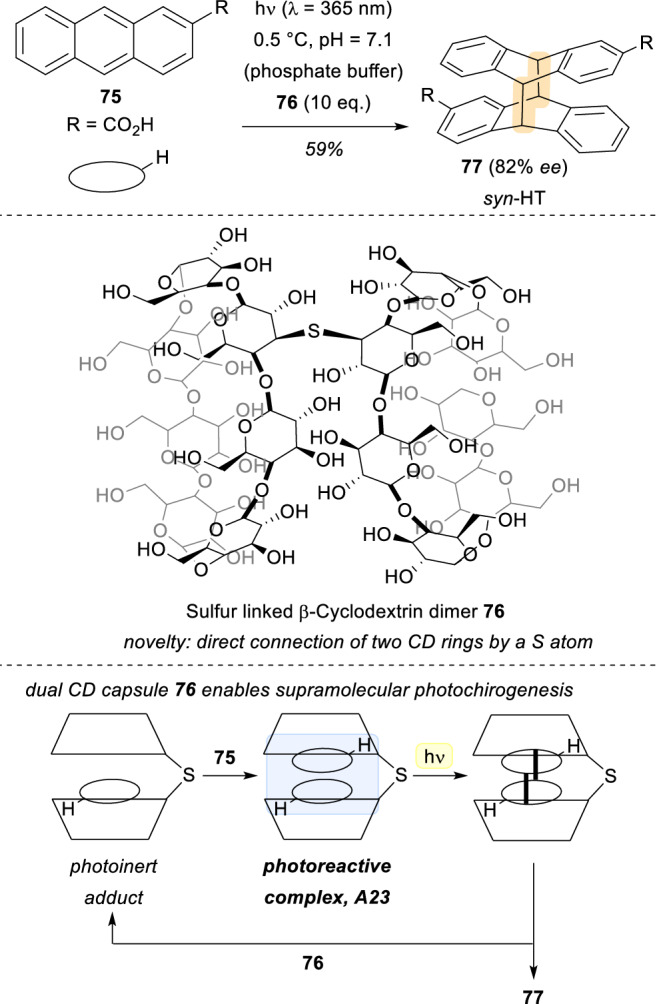
Novel sulphur-linked β-cyclodextrin dimers for the [4 + 4] photocycloaddition of AC

In a different scenario, β-cyclodextrins were employed in the last two decades as chiral molecular hosts for the efficient photocyclodimerization of anthracenecarboxylic acid **75** (AC). A lot of work has been done employing these molecules [57], and one of the latest examples on this field was reported in 2019 by Inoue and co-workers [58]. In this publication, a new class of cyclodextrin (CD) dimers **77** were designed where a sulphur atom connects two different saccharide units (Scheme 21). This novel structure is able to host two molecules of AC in a specific configuration thanks to multiple H-bonding interactions, creating a photoreactive complex **A23**, that is irradiated at λ = 365 nm for the [4 + 4]. photocycloaddition of two molecules of **75** to take place within the CD capsule. When comparing to other cyclodextrins, in this case the more confined, less flexible and better-defined CD capsule **76** is responsible for an assembly that grants great levels of stereocontrol in the transformation. Specifically, the *syn*-HT isomer **77** was delivered with an unprecedented enantiomeric excess (82%) in the field of AC photodimerizations with β-cyclodextrins.

**Scheme 22:**
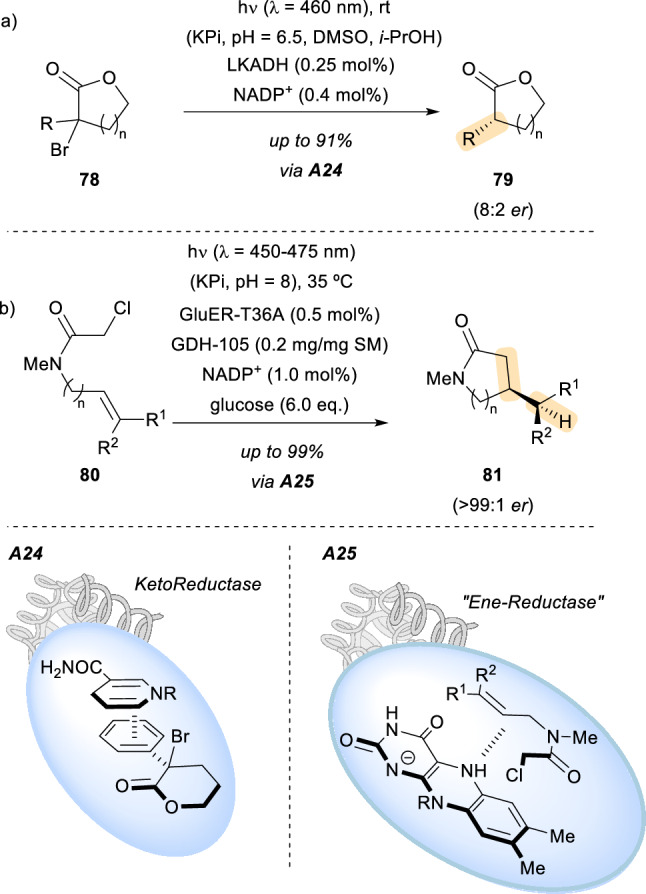
Examples of enzymes with photoactive cofactors employed in the development of photochemical reactions. *LKADH* lactobacillus kefiri-ADH, *GluER-T36A* gluconobacter oxydans ene-reductase-T36A, *GDH-105* glucose dehydrogenase, *NADP*^*+*^ nicotinamide adenine dinucleotide phosphate

Traditionally, the use of enzymes in synthetically relevant procedures has been limited to transformations that can be found in living beings [59]. However, in recent years some research groups have made a tremendous effort on the engineering of artificial proteins that aim not only to mimic these transformations, but also to enable new reactions through light irradiation employing these compounds [60]. The Hyster group has been working on the modification of the catalytic activity of enzymes, focusing their efforts on enzymes that depend on the activity of photoactive cofactors, using light to alter their properties [61]. In 2016, they reported that under irradiation with visible light, ketoreductase enzymes that depend on nicotinamide cofactors showed an enhanced catalytic activity [62]. In their natural environment, the ketoreductase’s catalytic activity is limited to reducing carbonyl functional groups. However, in the aforementioned publication by the Hyster group, ketoreductases were employed as initiators of radical species as well as a chiral source of hydrogen atoms. In particular, an enantioselective radical dehalogenation of lactones **78** was described (Scheme 22, a). Photoexcitation of the NADPH cofactor bound to the active site of the ketoreductase enzyme enabled the transformation of racemic α-halolactones to enantioenriched lactones **79** through the reduction of a prochiral lactone intermediate. The NADPH cofactor indeed behaves as both the photoreductant and the source of hydrogen atoms in this transformation. The cyclic lactones were obtained with good yields and in decent enantiomeric ratios (*er*). Essentially, the catalytic activity of this common cofactor in the excited state relies on the ability of the enzyme to bring the substrate and the cofactor together in the active site as shown in **A24**. Presumably, π–π interactions between the cofactor and the aromatic substituent of the substrate are responsible for the assembly and generation of a charge transfer complex.

Some years later, the same research group published an unprecedented stereoselective cyclization reaction of compounds such as **80** promoted by photoexcitation of flavoenzymes [63]. In analogy to the previous report, the flavin hydroquinone cofactor was excited with visible light enhancing the typical catalytic activity of the enzyme. In this case, the enzyme, an “ene”-reductase which typically is used for reduction reactions like other reductases, promotes an asymmetric radical cyclization upon visible light irradiation. For the reaction to occur, an assembly between the cofactor of the reductase enzyme and the substrate by means of weak interactions should occur. Then, the formation of a charge transfer complex **A25** leads, upon irradiation, to electron transfer and C − Cl bond cleavage, cyclization, and subsequent hydrogen atom transfer. This cyclization reaction delivered the products **81** in good to very good yields as well as with good enantiomeric ratio. However, the diastereomeric ratio did not show the same high values. The authors explained that this phenomenon arises from the double bond configuration of the substrates and their differing reactivity within the enzyme pocket. To prove their mechanistic hypothesis, a set of experiments with the (*E*)- and (*Z*)-isomers of the starting material were run. While the major diastereomer of the product was the same and was observed in similar *er* values, the diastereoselectivity was lower for the (*Z*)-isomer. According to the authors, this can be explained if the (*Z*)-alkene isomer has a presumably small degree of hydrogen atom transfer before the corresponding bond rotates. Thus, the enzyme favours the hydrogen atom transfer from one rotamer of the prochiral radical intermediate over the other, such that bond rotation becomes competitive.

The last selected examples of molecular assembly in a supramolecular environment concern micelles. Among the different topics of study with micelles, a particularly interesting research topic is the generation of hydrated electrons by using visible light. These hydrated electrons are strong reducing agents that can be generated in situ.

**Scheme 23:**
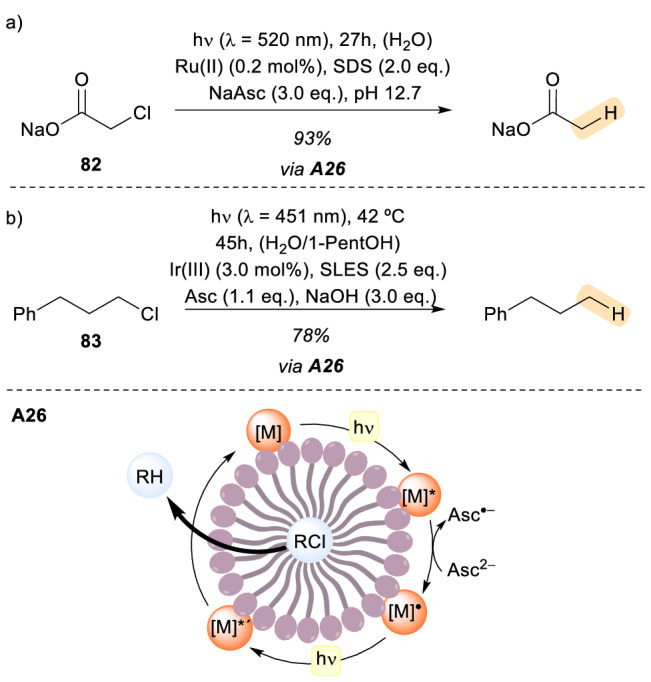
Micelle enabled dehalogenation of substrates under visible light irradiation. *Ru(II)* [Ru(bpy)_3_]Cl_2_·6H_2_O, *Ir(III)* [Ir(dtbby)(ppy)_2_](PF_6_)

In that regard, the group of Goez has been working on creating a robust and safe way of producing them in a laboratory-scale [64], using commercially available green LEDs. The energy of the green LEDs is not sufficient to trigger the liberation of a hydrated electron from any molecule, but this problem can be circumvented using a two-photon process analogous to the photosynthesis process (Scheme 23, A26). In this procedure, the redox catalyst responsible for absorbing the photon is a [Ru(bpy)_3_]^2+^ complex that is incorporated in a SDS (sodium dodecyl sulphate) anionic micelle. This catalyst gets excited upon irradiation, and after ISC to the metal-to-ligand charge-transfer triplet state of the catalyst, an electron is transferred from the sacrificial donor, in this case ascorbate ion. Then the one-electron-reduced catalyst ([M]^•^) absorbs a second photon, producing a hydrated electron and regenerating the catalyst in its ground state. This hydrated electron is then employed to cleave the C − Cl bond of substrate **82** [65]. In addition, the hydrated electrons were also used in other challenging processes such as the defluorination or hydrogenation of compounds which are normally inert towards photoredox processes employing visible light. The importance of the molecular assembly directed by the micelle is twofold: first, it enhances the natural lifetime of the excited state of the catalyst by increasing the energy difference between electronic states. Thus, the efficiency of the first electron transfer from the sacrificial donor, ascorbate, is improved. Secondly, it also stabilizes the one electron reduced form of the catalyst, suppressing the potential back-electron donation to ascorbate. However, water insoluble/lipophilic substrates, such as most alkyl chloride pollutants, proved to be resistant to this methodology. To address this challenge, the König research group developed a micellar system in which insoluble and non-activated alkyl chlorides **83** could participate in radical dehalogenation [66]. The authors focused their attention on iridium complexes as photocatalysts, since they act as stronger reducing agents. Specifically, the [Ir(dtbby)(ppy)_2_](PF_6_) complex was identified as the ideal photocatalyst for this purpose. This complex could form its one electron reduced species efficiently, in analogy to the previously described example, in an aqueous SLES (sodium lauryl oligoethylene glycol sulphate) solution. This catalyst-reduced species was also stabilized in this micellar environment. Moreover, the SLES micelle creates an environment that can solubilize the alkyl chloride substrate in a specific spatial arrangement. In summary, the assembly created by the micelle facilitates the C − Cl bond cleavage by bringing the stabilized reductive catalyst species and the substrate into close proximity. In addition, this method is employed to generate radical species from alkyl chlorides that can undergo addition and cyclization reactions.

## Conclusions and outlook

The growing attention in more sustainable and safe chemical processes has resulted in a vast devlopment of organic photocatalysis using visible light. With this Tutorial Review, we intended to describe and give visibility to the diverse varieties of molecular assemblies that are currently under development and looking for new reactivity using visible light as the main energy source. The formation of such assemblies allows for selectivity and control of the stereochemical outcomes in a wide set of different photoreactions. We have mainly focused on non-covalent interactions such as ion pairs, hydrogen bonds, van der Waals forces and π-effects as the forces that promote the creation of the molecular assemblies. The combination of different interactions in supramolecular assemblies was covered in the final section of this review, summarizing the most recent examples of the use of DNA, cages, enzymes and micelles in photoreactions. The development and the importance of new methodologies on such an emerging field is reflected in the novelty of the work gathered in this review.
